# Diversity and composition of gut protist in young rural Zimbabwean children

**DOI:** 10.3389/frmbi.2024.1399160

**Published:** 2024-05-22

**Authors:** Lorraine Tsitsi Pfavayi, Elopy Nimele Sibanda, Stephen Baker, Mark Woolhouse, Takafira Mduluza, Francisca Mutapi

**Affiliations:** ^1^ Institute of Immunology and Infection Research, School of Biological Sciences, University of Edinburgh, Edinburgh, United Kingdom; ^2^ Tackling Infections to Benefit Africa (TIBA) Partnership, University of Edinburgh, Edinburgh, United Kingdom; ^3^ Department of Pathology, National University of Science and Technology (NUST), Bulawayo, Zimbabwe; ^4^ TIBA Zimbabwe, Tackling Infections to Benefit Africa (TIBA) Partnership, University of Edinburgh, Edinburgh, United Kingdom; ^5^ Asthma Allergy and Immunology Clinic, Twin Palms Medical Centre, Harare, Zimbabwe; ^6^ Cambridge Infectious Diseases, Department of Medicine, University of Cambridge, Cambridge, United Kingdom; ^7^ Usher Institute of Population Health Sciences and Informatics, University of Edinburgh, Edinburgh, United Kingdom; ^8^ Department of Biochemistry, University of Zimbabwe, Harare, Zimbabwe

**Keywords:** gut protist, diversity, composition, Zimbabwe, children

## Abstract

**Background:**

The human gut microbiome harbours diverse species of archaea, bacteria, fungi, protists and viruses. To date, most gut microbiome studies have focused on bacteria, neglecting other microbial communities. Consequently, less is known about the diversity and abundance of the latter. Here, we aimed to characterise the diversity and composition of protists in the gut of preschool-aged children (PSAC) in rural Zimbabwe relative to host age, sex, and schistosome infection status.

**Methods:**

The gut protist of 113 PSAC (1–5 years) was examined via shotgun metagenomic sequencing and analysed for diversity. Variation in protist abundance with host and environmental factors was analysed by permutational multivariate analysis of variance (PERMANOVA). To investigate how the composition of specific taxa varies across age, sex, nutritional measures and *Schistosoma hematobium* infection status, analysis of the composition of microbiomes (ANCOM) was used.

**Results:**

Eighty protist genera were identified, and the most abundant genera detected was *Blastocystis.* The prevalence of pathogenic protists was comparatively low, with 12.4% and 3.4% of the participants’ gut colonised by *E. histolytica* and *Cryptosporidium*, respectively. Of all the independent variables only *S. haematobium* infection showed significant relationship with the structure of the gut protist, being associated with increases in *Peronospora*, *Pseudoperonospora*, *Plasmopara* and *Blastocystis* (FDR= 0.009).

**Summary:**

This study provides data on the prevalence and diversity of the gut protists in young Zimbabwean children with an emphasis on the host factors; age, sex and schistosome infection status. Our results showed no association between the host factors investigated, including anthropometric measures adjusted for age and the intestinal protist composition and structure, but *S. haematobium* infection status was associated with composition of specific taxa. There is a need for more studies determining how pathogenic protist interact with non-pathogenic protist in people exhibiting clinical symptoms to inform therapy and nutraceuticals.

## Introduction

The gut microbiome harbours diverse species of archaea, bacteria, fungi, protists and viruses that contribute to host biology (Garcia-Bonete et al., 2023). To date, gut microbiome studies in humans have focused on bacteria neglecting other microbial communities such as protists (Vargas-Albores et al., 2023). There are at least fifteen different protist genera from diverse groups that either parasitise or are commensals, in the human gut (Hamad et al., 2016) and thus, there is a need to study the relative importance of these in human health. Gut protists show high inter-individual variability but less abundance and diversity than bacteria (Hooks and O’malley, 2020), and research has primarily centred on disease-causing parasitic protists. However, gut protists are often commensals and their role in the gut remains poorly understood. It has been suggested that some commensal species induce beneficial host innate immune responses (Underhill and Iliev, 2014), as well as other potential benefits such as eubiosis and alleviating the symptoms of inflammatory bowel disease (IBD), Crohn’s disease and Type 1 diabetes (T1D) (Parfrey et al., 2014; Lukeš et al., 2015; Bach, 2018; Ianiro et al., 2022). Furthermore, some protists such as *Blastocystis* and non-pathogenic *Entamoeba* have been associated with a healthy gut (Audebert et al., 2016; Chabé et al., 2017). Nonetheless, the impact of these commensals and the pathogenic nature of parasitic protists remains poorly studied. Pathogenic protists such as *Giardia duodenalis*, *Entamoeba histolytica* and *Cryptosporidium* spp (Maas et al., 2014). are important for human health as they are widely distributed and cause serious pathology. The World Health Organization (WHO) has identified *Cryptosporidium* spp. as the most common diarrhoea-causing protist globally (Pedersen et al., 2014), and giardiasis, caused by *Giardia duodenalis* is experienced by 20–30% of people in developing countries (Hajare et al., 2022). Accordingly, both parasites *Cryptosporidium* spp. and *G. duodenalis* were included in the “Neglected Disease Initiative” launched by the WHO in 2004 (Osman et al., 2016). Amoebiasis is also highly prevalent with amoebic colitis, estimated to kill more than 55 000 people each year (Lozano et al., 2012). These numbers underscore the need to address protist-related diseases. Embracing the One Health approach (Galán-PuChades et al., 2021; Massengo et al., 2023) is essential for comprehensively understanding and effectively combating these parasitic protists. By recognising the multifaceted nature of these infections and their implications across different domains, we can develop holistic strategies to tackle the challenges posed by these neglected diseases.

In Africa, the exact burden of these protist infections is difficult to quantify, and reports can be affected by geographic region, study design, sample size, incubation, symptom severity, and the sensitivity of the diagnostic modality used (Shirley et al., 2018). Furthermore, diagnostic capacities and surveillance are often limited in this continent. The infections, which are caused by these protists are characterised by chronic to severe diarrhoea, sometimes accompanied by abdominal cramping, flatulence, nausea, vomiting, anorexia, fatigue, low-grade fever; some even cause malabsorption as well as severe debilitating illness, especially in immunosuppressed populations (Stark et al., 2009; Kucerova et al., 2011; Stensvold et al., 2011). The resulting intestinal malabsorption can be so severe that chronic infections in children can be associated with retardation of growth and development (Farthing, 2006), especially in developing countries where hygienic and healthcare standards (Wawrzyniak et al., 2013; El Safadi et al., 2014; Lhotská et al., 2020) are continuously deteriorating. More than 82% of all under-five deaths in Africa are caused by diarrhoeal diseases (Gupta, 2012). In 2015, diarrhoea was the second leading cause of childhood deaths in Zimbabwe, contributing to 10% to 15% of deaths in under-five children (Tate et al., 2016; UNICEF, 2016). However, despite several years of policy and practice aiming to reduce the incidence, morbidity and mortality associated with diarrhoea in the country, it remains a significant contributor to under‐five morbidity and mortality (Mukaratirwa et al., 2018). *G. duodenalis* and *E. histolytica* have been shown to be associated with differences in the composition and diversity of gut microbiota (Burgess and Petri, 2016; Barash et al., 2017; Beatty et al., 2017). Given that these protists are known to cause disease in certain individuals and that the reported results are confounded by disease status (asymptomatic/symptomatic), it is unknown whether the observed changes in microbial composition are due to the protist or ongoing inflammation (Nieves-Ramírez et al., 2018).

Over the past decade there has been a growing interest in investigating the diversity of microbiome in the healthy human population, partly due to the hypothesis that one or more of these genera/species could be conducive to human health (Lukeš et al., 2015; Chabé et al., 2017; Stensvold and van der Giezen, 2018). However, little is known about the prevalence and factors that influence the abundance and diversity of gut protists in humans (Mann et al., 2020). Thus, the aim of this study was to characterise the gut protist using shotgun metagenomics that may be associated with human health, especially in Zimbabwe where dysentery and diarrhoeal diseases are the cause of significant childhood mortality. We further related this to host factors, specifically; we were interested in the following host factors: age, sex and schistosome infection status. Moreover, this study aimed to provide additional data on the gut microbiome studies in Southern Africa.

## Materials and methods

### Ethical approval and consent

The study received ethical and institutional approval from the Medical Research Council of Zimbabwe (MRCZ/A/1964) and the University of Edinburgh respectively. Permission to conduct the study was obtained from the Mashonaland Central Provincial Medical Director. Prior to enrolment, the study aims and procedures were explained to all participants and their parents/guardians in their local language (Shona). The parents/guardians of the participants provided written informed consent and participation in the study was voluntary, with participants able to withdraw at any time.

### Study design, population and site

This cross-sectional study was conducted in Shamva district, one of the seven districts in the Mashonaland Central province of Zimbabwe. It was part of a larger research project, the ‘Paediatric Schistosomiasis Study’, which investigated the overall health impact of paediatric schistosomiasis in children aged 5 years and below; commonly called pre-school aged children (PSAC) (Osakunor et al., 2020; Pfavayi et al., 2021).

A cohort of 116 children was selected from the baseline survey for microbiome analysis (Osakunor et al., 2020). Of these, 113 met the inclusion criteria for the current study. The criteria were as follows: a) they had to be lifelong residents of the study area; b) no current episode of diarrhoea or dysentery; c) their guardian/parent had to consent to their participation; d) there had to be socio-demographic data available; and e) consent for their stool samples to be used for microbiome characterisation. The 3 excluded children had missing metadata and could not be included in downstream analysis.

### Sample collection, processing and DNA extraction

Urine and stool samples were collected from participants to screen for schistosomiasis and soil-transmitted helminths, using methods previously described by our group (Osakunor et al., 2020). Briefly, about 50 ml of urine was collected on three successive days and a stool sample was collected on a single day from each participant. For young children, urine bags (Hollister 7511 U-Bag Urine Specimen Collector, Hollister Inc., Chicago, IL, USA) and disposable diapers were used for collection. Urine samples were analysed for *Schistosoma haematobium* using the standard urine filtration method, while stool samples were examined for *Schistosoma mansoni* using the Kato-Katz technique.

Stool DNA for gut microbiome analysis was extracted from the samples using the QIAamp DNA Stool Mini Kit (QIAGEN), following the manufacturer’s protocol. DNA purity was verified using a Qubit fluorometer (Thermo Fisher Scientific) at the University of Edinburgh before DNA sequencing. The DNA samples were shipped on dry ice for library preparation and shotgun metagenomic sequencing to BGI (Beijing Genomics Institute, Shenzhen, China).

### Next-generation sequencing

The preparation of DNA samples for next-generation sequencing (NGS) was carried out as previously outlined (Osakunor et al., 2020). FASTQC and BBduk2 [BBMap—Bushnell B.—https://sourceforge.net/projects/bbmap/] were utilised for sequencing quality control and trimming of the reads, respectively. The trimmed reads were used as input to align directly to reference sequence databases downloaded via NCBI GenBank clade-specific assembly summary.txt files (ftp:/ftp.ncbi.nlm.nih.gov/genomes/genbank) utilising k-mer alignment (KMA) (Osakunor et al., 2020; Pfavayi et al., 2021). To determine the biome content of each sample, a classification was performed on the read pairs using Kraken software (version 2.1.1; https://github.com/JenniferLu717/KrakenTools 2 which includes RefSeq archaea, bacteria, viruses, plasmid complete genomes, UniVec Core, and the human reference genome, GRCh38). Paired reads were classified using the entire set of reads for each and a kraken-report was generated to provide taxonomic information. After classification, reads identified as belonging to the human genome were filtered out. Based on the obtained taxon ID, a putative taxonomy was assigned to the obtained primary alignment of mapped sequences. Taxon IDs and associated taxonomy classifications were obtained from reference microbial genomes downloaded from NCBI (ftp:/ftp.ncbi.nih.gov/pub/taxonomy/taxdump.tar.gz), and then assigned to all taxonomic levels. To obtain information about the abundances of features in the datasets relative to each other, datasets were treated as compositional and prior to transformations a small pseudo-count of half the smallest non-zero abundance per feature was added to each feature for each of the normalised abundance matrices. Centred log ratio (clr) transformations were performed on the microbiota abundance tables and for all subsequent analysis, these clr matrices were used (Osakunor et al., 2020; Pfavayi et al., 2021).

### Data analysis

Statistical analyses and data visualisation were performed using various Bioconductor packages within the R environment v3.6.1. Using R, the Euclidean distances were calculated to determine if sample-related metadata predicted within-group microbiome dispersion. The effect of such metadata on sample dissimilarities was analysed using permutational multivariate analysis of variance (PERMANOVA; adonis2 function in the vegan package) with a significance threshold of P < 0.05. A false discovery rate (FDR (Benjamini–Hochberg FDR)) correction was applied to counteract multiple testing (Benjamini and Hochberg, 1995). The analysis of composition of microbiomes (ANCOM) (Mandal et al., 2015) was employed to investigate how the composition of specific taxa varies across statistically significant metadata (from PERMANOVA) while controlling for other variables of interest. Box plots stratified by specific independent variables, using the clr-transformed abundance data of significant taxa previously identified by ANCOM were used to highlight differences in groups. Bar plots from normalised and zero-corrected abundance matrices were used to give an overview of the microbiota gene abundances across all samples. Beta diversity was measured using the Bray-Curtis index and Principal coordinate analysis (PCoA) ordination.

## Results

### Population demographics

The mean age of the 113 study participants was 3.7 ± 1.1 years, with 57 (50.4%) males and 56 (49.6%) females. Of these 113 children, 18 were *S. haematobium* positive. The demographic information of the study participants are compiled in **Table 1**.

**Table 1:** Demographic characteristics of study population.

Demographic categories			Frequency	Percentage (%, 95% CI)
	n			
**Gender**	113	Female	56	49.6 (40.0 - 59.1)
		Male	57	50.4 (40.8 - 60.0)
**Age group (years)**	113	≤3	69	61.1 (51.4 - 70.1)
		4	29	25.7 (17.9 - 34.7)
		5	15	13.3 (7.6 - 21.0)
***S.haematobium* infection status**	113	Negative	95	84.1 (76.0 – 90.3)
		Positive	18	15.9 (9.7- 24.0)
Nutritional and growth factors				
**Breastfed (months)**	90	<6	1	1.1 (0.03 - 6.0)
		≥ 6	89	98.9 (94.0 - 100.0)
**Solid food introduction (months)**	102	<6	32	31.4 (22.6 - 41.3)
		≥ 6	70	68.6 (58.7 - 77.5)
**Stunted (HAZ)**	109	Yes	16	14.7 (8.6–22.7)
		No	93	85.3 (77.3–91.4)
**Malnourished (WHZ)**	107	Yes	4	3.7(1.0–9.3)
		No	103	96.3(90.7–99.0)
**Malnourished (MUACZ)**	100	Yes	8	8.0(3.5–15.2)
		No	92	92.0(84.8–96.5)
**Underweight (WAZ)**	108	Yes	6	5.6 (2.1–11.7)
		No	102	94.4(88.3–97.9)
**Total**			**113**	**100**

Classification of nutritional status was based on a cut off <−2 Z scores. WHA, weight-for height Z scores; HAZ, height-for-age Z scores; MUACZ mid-upper arm circumference z score; WAZ, weight-for-age.

### Characterisation of the intestinal protist microbiota

**Figure 1:**
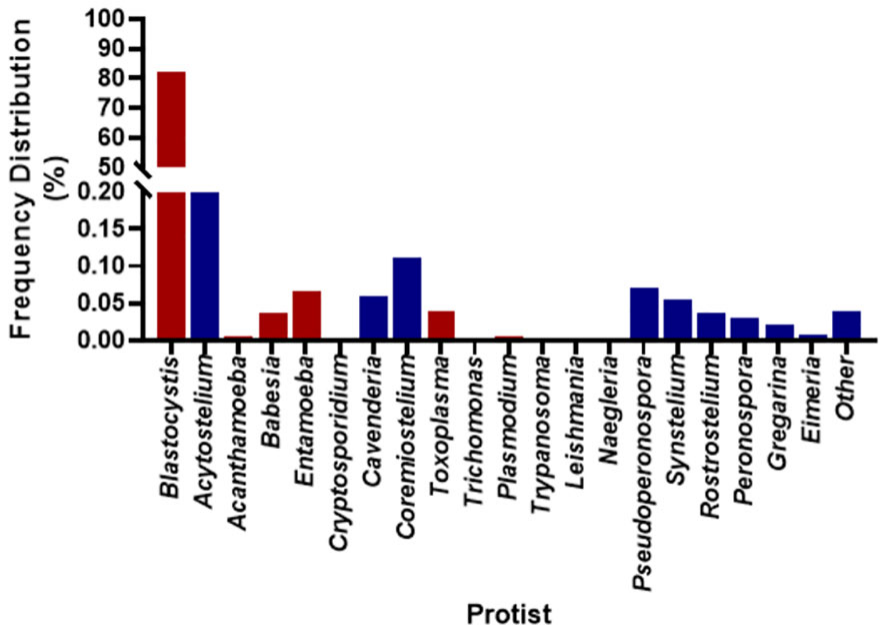
Overall prevalence of protist detected in the 113 samples. Red coloured bars are pathogenic protist, and blue coloured bars are non-pathogenic protist.

There were between 3,994,704 and 13,164,482 classified read pairs per sample. At any taxonomic level, at least 33% of the mapped read pairs could not be assigned a taxonomic classification and were therefore classified as “unknown”. Overall, 80 protist genera were detected in all 113 samples and were dominated by *Blastocystis*, representing 82% of the total protist community (**Figure 1**). Seven *Blastocystis* subtypes were identified in the study, ST1, ST2, ST3, ST4, ST6, ST8 and ST9 (**Table 2**). ST2 and ST3 were the most prevalent subtype, found in all PSAC (100%) followed by ST1 and ST6 in 110/113 PSAC (97.3%). *E. histolytica* was detected in 14/113 (12.4%), while *E. dispar* was detected in 90/113 (79.6%) of PSAC.

**Table 2:** *Blastocystis* subtypes and *Entameoba* species distribution.

Blastocystis subtype	Distribution (%)
**ST1**	97.3
**ST2**	100
**ST3**	100
**ST4**	18.1
**ST6**	97.4
**ST8**	66.4
**ST9**	74.1
Entamoeba species	
***Entamoeba histolytica***	12.4
***Entamoeba dispar***	79.6
***Entamoeba moshkovskii***	1.7
***Entamoeba nuttalli***	68.9

**Figure 2:**
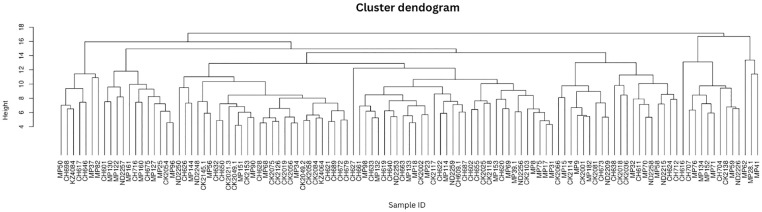
Protist composition dendrograms. Clustered dendrograms show protist genera per sample. The vertical axis of the dendrogram represents the distance or dissimilarity between clusters. The horizontal axis represents the population and clusters.

The protist genera showed homogeneity with no distinct clustering according to metadata (**Figure 2**) which indicates a high level of diversity in the cohort.

### Relative abundance of protist genera in the gut microbiome

**Figure 3:**
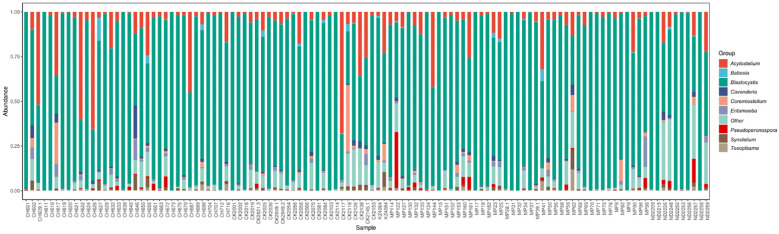
Overview of the protist microbiota abundance and diversity. Stacked bar charts show the most abundant protist genera per sample (*n*=113), proportional to the total microbiota within each sample. “Other” denotes abundance data for all other taxa in the abundance data set.

**Figure 4:**
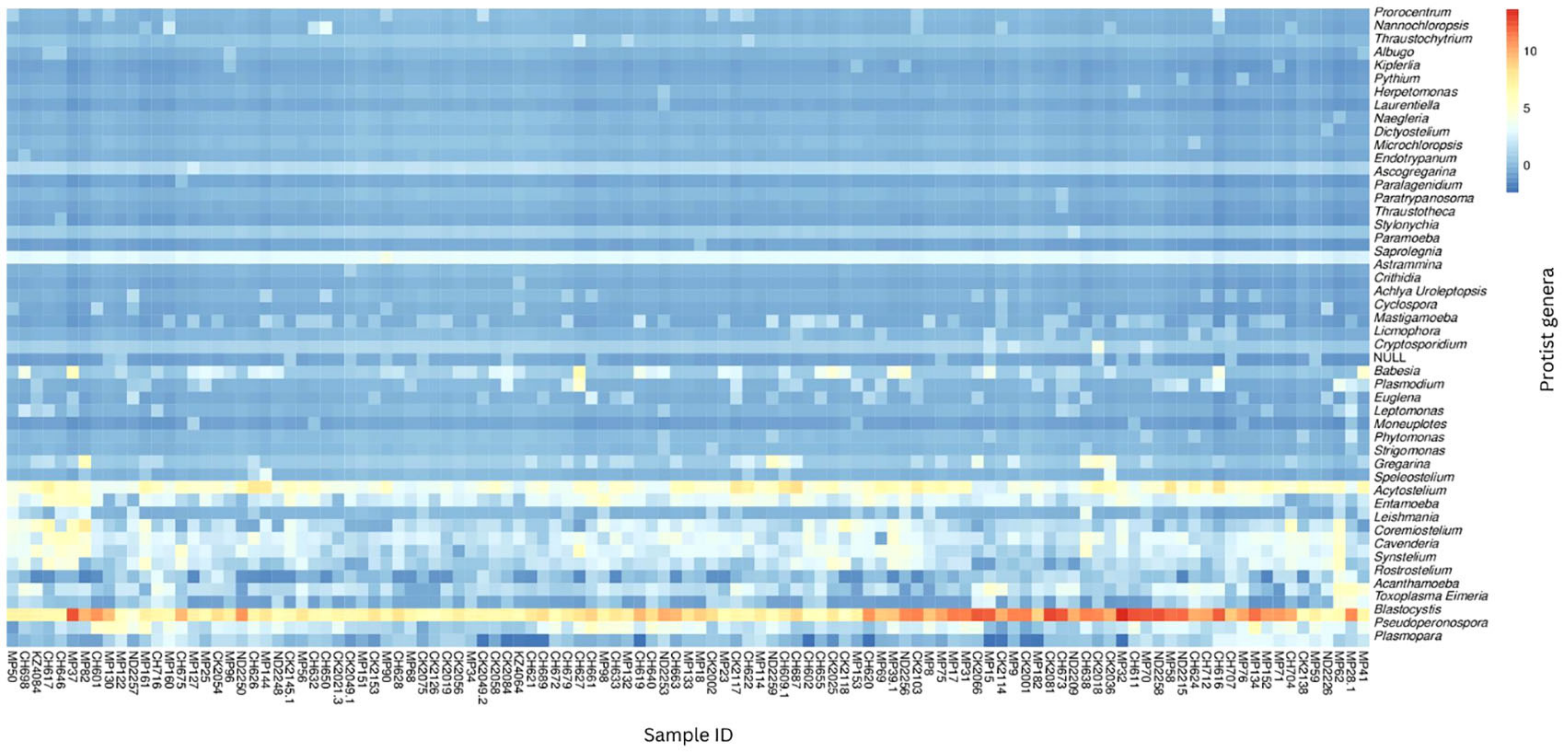
Heat map of protist composition of study population. The relative abundances of the protist genera identified in the study population are depicted on a heat map. The row-scaled relative abundance of each taxon across all samples is represented by the hue (blue to red) of the heat map.

Abundance was calculated for each microbial taxon across all samples. The most prevalent genera was *Blastocystis* (100%, present in all the samples). Majority of the detected phyla were classified as “Unknown”, as putative classification could not be assigned to at least 33% of the mapped read pairs. **Figure 3** depicts a summary of the nine most abundant protists in the study population, while **Figure 4** depicts a composition heat map of all the protists detected.

### Variation in the protist and association with participant metadata

#### Principal coordinate analysis

**Figure 5:**
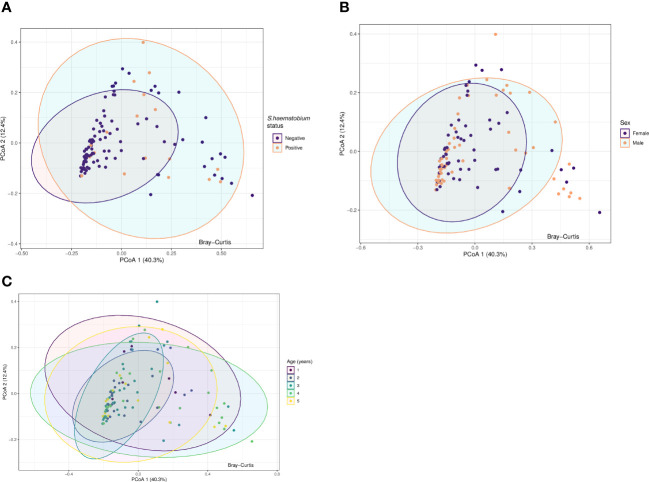
Principal coordinate analysis (PCoA) plots for protists microbiota across samples, annotated by schistosome status **(A)**, sex **(B)** and age (years) **(C)**. Results of Principal-component analysis (PCoA) showing positions of all samples along the first two axes (explaining 40.3% and 12.4% of the total variation respectively). The ordination was based on Bray-Curtis dissimilarities. The eigenvalues associated with the eigenvectors are used to describe the amount of explained variation per axis. Ellipses represent the confidence intervals (CIs) at 95%.

Principal Coordinate Analysis (PCoA) was used to examine variability and patterns in the data set across the first two principal components. PCoA explained 52.7% of the total variance between the samples. We were unable to distinguish the samples by *S.haematobium* infection status, sex or age along the first or second axis (**Figure 5**) suggesting the presence of communities with similar overall compositions.

#### Association between gut protist and participant metadata

To test whether the sample-related metadata (age (categorised based on individual ages), sex, nutritional status and *S. haematobium* infection status) were different with respect to centroid and dispersion, PERMANOVA analysis was performed. PERMANOVA analysis showed a significant effect of *S. haematobium* infection status (FDR= 0.009) across the samples, signifying that differences exist between S*.haematobium* infection status groups. **Table 3** provides an overview of the results of the analysis.

**Table 3:** Summary of sample metadata and association with gut protist microbiome.

	n	P-value	FDR
**Sex**	113	0.184	0.361
**Age_years**	113	0.089	0.267
**Malnourished_(WHZ)**	107	0.440	0.659
**Malnourished (MUACZ)**	100	0.683	0.769
**Underweight (WAZ)**	108	0.201	0.361
**Stunted (HAZ)**	109	0.056	0.252
***S.haematobium* status**	113	0.001	0.009
**Months_breastfed**	90	0.785	0.785
**Months_Solid_food**	102	0.636	0.769

Classification of nutritional status was based on a cut off <−2 Z scores. WHA, weight-for height Z scores; HAZ, height-for-age Z scores; WAZ, weight-for-age Z Score; MUACZ; mid-upper arm circumference z score; p-value-unadjusted p-value; FDR- adjusted p-value (FDR-corrected).

#### Gut protist analysis by *S.haematobium* infection

From the PERMANOVA results, further analysis via ANCOM showed that the abundance of four specific protist genera was associated with *S.haematobium* infection.

**Figure 6:**
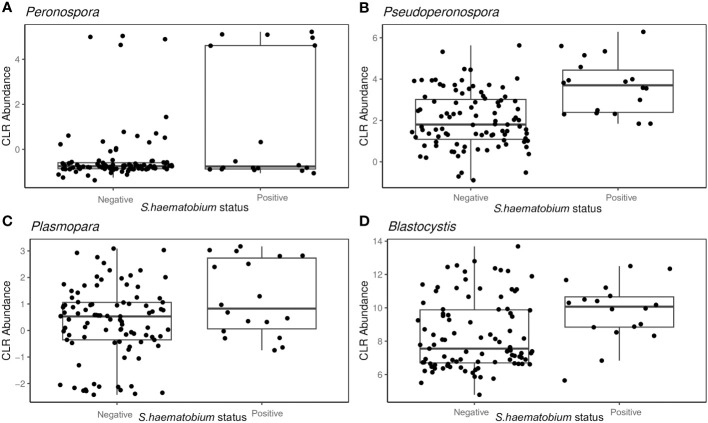
Box plots showing the abundance of specific protist genera Peronospora **(A)**, Pseudoperonospora **(B)** Plasmopara **(C)** and Blastocystis **(D)**, grouped by S. haematobium infection status. The horizontal box lines represent the first quartile, the median, and the third quartile. Whiskers denote the range of points within the first quartile −1.5× the interquartile range and the third quartile +1.5× the interquartile range.

*Peronospora* (W= 28), *Pseudoperonospora* (W= 28), *Plasmopara* (W= 26) and *Blastocystis* (W= 25), showed variation with *S.haematobium* infection. The magnitude of the differences in abundance between groups are shown in **Figure 6**. The abundance of all the identified genera that varied with *S.haematobium* infection was slightly higher in schistosome-positive children.

## Discussion

To date, diarrhoeal disease is the world’s second leading cause of death among young children in developing countries (Hartman et al., 2022; Benzamin and Hoque, 2024). While a range of different pathogenic organisms can cause diarrhoea, including viruses and bacteria (Wilhelmi et al., 2003; Al-Thani et al., 2013; Najafi et al., 2013; Weam et al., 2016), a significant percentage of cases of paediatric diarrhoea is associated with the presence of gut protists; especially in developing countries where declining hygienic standards and poor healthcare standards (Wawrzyniak et al., 2013; El Safadi et al., 2014; Lhotská et al., 2020) are a persistent problem.

In the current study, we characterised the diversity and composition of protists in the gut of preschool-aged children (PSAC) in rural Zimbabwe in relation to host age, sex and schistosome infection status. The overall prevalence of any enteric protist in the population was 100%, and some individuals had multiple colonisation. *Blastocystis* was detected in all the one hundred and thirteen children (100%), which agrees with the results obtained by others (El Safadi et al., 2014; Iebba et al., 2016; Poulsen et al., 2016). Overall, ten different *Blastocystis* subtypes (ST1–ST9 and ST12) have been found in humans (Alfellani et al., 2013; Stensvold et al., 2020), of which we detected seven in this study, specifically ST1–ST4, ST6, ST8 and ST9. Our results on the subtype distribution across humans are in agreement with findings from similar studies (El Safadi et al., 2016). However, in humans, the most frequently detected subtypes are ST1-ST3 or ST1-ST4 (Scanlan et al., 2014, 2016; Jalallou et al., 2017; Mohammad et al., 2017; Valença Barbosa et al., 2017). In the present study, the most common subtype were ST2 and ST3.

Despite its high prevalence, the role of *Blastocystis* in health and disease remains controversial because asymptomatic colonisation is common, as demonstrated by the higher prevalence in healthy controls (Andersen and Stensvold, 2016; Kurt et al., 2016; Lhotská et al., 2020); however, some studies have provided evidence for *Blastocystis*-induced pathogenicity (Wawrzyniak et al., 2013). It has also been argued that identification of *Blastocystis* from patient samples is not clinically significant but should be used as a marker of potential exposure to other pathogenic protists.

It is important to highlight that at the time of sample collection, none of the children were suffering from diarrhoea/dysentery nor presented with intestinal symptoms which could have decreased the probability of pathogenic protist detection. However, it was important to determine the non-pathogenic protist presented in the children’s gut microbiome since their existence points to a faecal-oral transmission in infected people. Furthermore, the presence of these non-pathogenic parasites is an indicator of the sanitary and health conditions in a particular place (Sarkari et al., 2016).

Among the *Entamoeba* species infecting humans, *E. histolytica* is so far the only one associated with amoebiasis (Kantor et al., 2018). Amoebiasis is one of the most problematic parasitic infections worldwide, particularly in poor communities from developing countries, resulting in severe conditions such as amoebic colitis and amoebic liver abscess and even in fatal cases (Roure et al., 2019; Shirley et al., 2019). In the current study, while *E. dispar* was detected in ninety (79.6%) of participants, *E. histolytica* was detected in fourteen participants (12.4%). Similarly, there was a low infection frequency of *Cryptosporidium* with a 3.4% prevalence. As previously mentioned, none of the children had intestinal symptoms in this study, which could explain the observed values. Here, *G. duodenalis* or *Dientamoeba fragillis* infections were undetected, yet in a previous study in Zimbabwe the prevalence of *G. duodenalis* was high and highly prevalent in the urban areas (Mason et al., 1986). Possible reasons for this difference could have been due to sampling methods, differences in study population or prevalence of the parasites. In a recent multicentre birth cohort study in eight low income countries *Cryptosporidium* spp. were some of the top diarrhoea-associated pathogens (Korpe et al., 2018).The evaluation of individuals with no intestinal symptoms could have resulted in the difference observed between our results and the Korpe et al (Korpe et al., 2018).

In the current study we also detected a number of *Acanthamoeba* spp., which according to our knowledge have not been reported in Zimbabwe. However, *Acanthamoeba* spp. are known to be widely distributed in the environment and have been found in many countries worldwide, including Africa. They are ordinarily free living within the environment but capable of causing severe infections under suitable circumstances. In addition to increasing cases of *Acanthamoeba*-associated infections globally (Carnt et al., 2018; Randag et al., 2019; Scruggs et al., 2019; Höllhumer et al., 2020) this finding warrants further investigations to identify the risk factors associated with exposure and subsequent infection. Such studies will be essential for understanding the epidemiology of the organism, implementing appropriate public health measures.

Among the host factors evaluated in our study, only *S. haematobium* infection showed a significant association with intestinal protist colonisation. We observed that the protist were largely heterogeneous, with *Peronospora, Pseudoperonospora*, *Plasmopara* and *Blastocystis* clearly differentiating the microbiome of schistosome-infected versus uninfected children. Kay et al (Kay et al., 2015), and Schneeberger et al (Schneeberger et al., 2018), reported that schistosome infection is associated with alterations in the diversity and abundance of specific taxonomic groups in the microbiome, as was observed in the current study. However, it was beyond the scope of this study to determine the causal relationship between protist composition and schistosome infection. As *Peronospora, Pseudoperonospora and Plasmopara* are plant pathogenic oomycetes, and are obligate aerobes that cannot colonise the human gut (Savory et al., 2011; Thines and Choi, 2015; Koledenkova et al., 2022). It is most likely that these are transient members of the gut and not resident members and hence their presence might not have an effect on the gut microbiome composition. Nonetheless, it is possible that schistosome infection resulted in alterations of the gut microbiome favouring a particular structure of gut protists or the presence of specific protists predisposed the children to schistosome infection, for example by influencing the innate immune responses (Kay et al., 2015).

We investigated the association between schistosomiasis and pathogenic intestinal protists because of its significant public health implications. For instance, both infections are common in developing countries where access to clean water and sanitation is limited, resulting in co-infections (Blackwell et al., 2013). Thus, individuals infected with both schistosomiasis and an intestinal protists could be at risk for more severe symptoms and complications. Furthermore, schistosomes and pathogenic intestinal protist can interfere with each other; for example, *Schistosoma mansoni* infection is known to affect the gut microbiome (Floudas et al., 2019) and this can affect the susceptibility or resistance of the host to other pathogens.

Likewise, age is an important factor in colonisation with intestinal protists with young children being more susceptible to infection and as the protists can contribute to malnutrition and stunting in children by interfering with the absorption of nutrients (Mondal et al., 2006; Mulatu et al., 2015). We further assessed the association between age, growth standards and intestinal protists. In our study, there were no significant differences in stunting, malnutrition or age. This could have been due to the small sample size of stunted or malnourished children. Furthermore, there were no sex-related differences in the children’s gut protist. Given that all the children enrolled in this study were ≤ 5 years, it was not surprising that there were no sex-related differences, as they have not reached puberty, where the influences of the sex hormones on physiology or innate immune responses can be marked (Kay et al., 2015). Furthermore, this finding implies male and female children have equal chances of colonisation since they are engaged equally in all activities around that age.

Despite ongoing efforts to enhance disease surveillance and response, many African countries face challenges in accurately diagnosing and reporting infectious diseases due to the remoteness of some communities, shortage of skilled health care workers and laboratory facilities (WHO, 2015). This has resulted in most of these diseases being neglected (Bär et al., 2015). The current study was carried out in a rural area where most of the community members are subsistence farmers, growing crops and raising livestock. Therefore as previous studies have shown that numerous animal species including livestock are infected with some of these intestinal protist, a ‘One Health’ approach to intestinal protist prevention, surveillance, monitoring and control, should be adopted broadly (Yanyan et al., 2022). Adopting the One Health approach enables integration of data from various disciplines and researchers can gain a comprehensive understanding of disease dynamics (Bhatia, 2019; Nzietchueng et al., 2023). Such collaborative work could potentially facilitate early detection and response to emerging diseases in both humans and animals, preventing the spread of potential epidemics. Additionally, this could potentially enhance the implementation of evidence-based interventions, ultimately leading to better research outcomes (Mackenzie and Jeggo, 2019).

The present study is subject to limitations that should be acknowledged and considered in the interpretation of its findings. Firstly, a key limitation arises from the reliance on reference databases primarily tailored for prokaryotes in the taxonomic assignment process of shotgun metagenomics. Consequently, these databases may not encompass a comprehensive representation of eukaryotic organisms, which in turn poses challenges in accurately identifying and classifying eukaryotic taxa (Lind and Pollard, 2021). This inherent limitation can result in reduced taxonomic resolution and potentially lead to misinterpretations regarding the composition of the eukaryotic microbiome. Furthermore, the eukaryotic microbiome exhibit lower abundance compared to its prokaryotic counterpart (Lu and Salzberg, 2018; Steinegger and Salzberg, 2020). The pronounced presence of prokaryotic DNA can overshadow the detection of less abundant eukaryotic DNA, thereby introducing a bias into the analysis. Consequently, there is a potential risk of underestimating the diversity and functional potential of the eukaryotic microbiome.

## Conclusion

The current study characterised the intestinal protist in rural Zimbabwean children. Our results showed that there was no association between the host factors investigated and the intestinal protist composition. However, *S. haematobium* infection status was associated with composition of specific taxa. Although the prevalence of pathogenic protist was relatively low, there is need for further research to investigate the interactions between pathogenic and non-pathogenic protists in individuals displaying clinical symptoms, in order to provide insights for the development of therapeutic interventions and nutraceuticals.

## Data availability statement

Publicly available datasets were analysed in this study. This data can be found here: https://www.ncbi.nlm.nih.gov/bioproject/?term=PRJNA521455. Sequence Read Archive (SRA) of the National Centre for Biotechnology Information (NCBI) database under the BioProject accession number PRJNA521455.

## Ethics statement

The studies involving humans were approved by Medical Research Council of Zimbabwe (MRCZ/A/1964) and the University of Edinburgh. The studies were conducted in accordance with the local legislation and institutional requirements. Written informed consent for participation in this study was provided by the participants’ legal guardians/next of kin.

## Author contributions

LP: Data curation, Formal analysis, Investigation, Writing – original draft, Writing – review & editing. ES: Supervision, Writing – review & editing. SB: Supervision, Writing – review & editing. MW: Supervision, Writing – review & editing. TM: Conceptualization, Investigation, Writing – review & editing. FM: Conceptualization, Formal analysis, Investigation, Writing – review & editing.
